# Watermelon‐Derived Extracellular Vesicles Influence Human Ex Vivo Placental Cell Behavior by Altering Intestinal Secretions

**DOI:** 10.1002/mnfr.202200013

**Published:** 2022-08-19

**Authors:** Kate Timms, Beth Holder, Anil Day, John Mclaughlin, Karen A. Forbes, Melissa Westwood

**Affiliations:** ^1^ Maternal and Fetal Health Research Centre School of Medical Sciences University of Manchester Manchester M13 9WL UK; ^2^ Manchester University NHS Foundation Trust Manchester Academic Health Sciences Centre Manchester M13 9WL UK; ^3^ Department of Metabolism Digestion and Reproduction Institute of Reproductive and Developmental Biology Imperial College London London UK; ^4^ Division of Molecular and Cellular Function School of Biological Sciences University of Manchester Manchester M13 9PT UK; ^5^ Division of Diabetes Endocrinology and Gastroenterology University of Manchester Manchester M13 9PT UK; ^6^ Department of Gastroenterology Salford Royal NHS Foundation Trust Salford M6 8HD UK; ^7^ Discovery and Translational Science Department Leeds Institute of Cardiovascular and Metabolic Medicine Faculty of Medicine and Health University of Leeds Leeds LS2 9JT UK

**Keywords:** FGR, interkingdom communication, plant, pregnancy, trophoblast

## Abstract

**Scope:**

During pregnancy, mother‐to‐fetus transfer of nutrients is mediated by the placenta; sub‐optimal placental development and/or function results in fetal growth restriction (FGR), and the attendant risk of stillbirth, neurodevelopmental delay, and non‐communicable diseases in adulthood. A maternal diet high in fruit and vegetables lowers the risk of FGR but the association cannot be explained fully by known macro‐ and micronutrients.

**Methods and results:**

This study investigates if dietary‐derived extracellular vesicles (EVs) can regulate placental function. The study characterizes the microRNA and protein cargo of EVs isolated from watermelon, show they are actively internalized by human intestinal epithelial cells in vitro, use mass spectrometry to demonstrate that they alter the intestinal secretome and bioinformatic analyses to predict the likely affected pathways in cells/tissues distal to gut. Application of the watermelon EV‐modified intestinal secretome to human placental trophoblast cells and ex vivo tissue explants affects the trophoblast proteome and key aspects of trophoblast behavior, including migration and syncytialization.

**Conclusion:**

Dietary‐derived plant EVs can modify intestinal communication with distal tissues, including the placenta. Harnessing the beneficial properties of dietary‐derived plant EVs and/or exploiting their potential as natural delivery agents may provide new ways to improve placental function and reduce rates of FGR.

## Graphical abstract

(A) The content of watermelon extracellular vesicles (EVs) differs from watermelon cells. (B) Plant EVs are known to survive digestion; here we show (C) they are internalised into intestinal epithelial cells in vitro, (D) resulting in an altered basal secretome (representing post‐digestion circulation), and beneficial changes to placental function in vitro: increased (E) syncytialisation and (F) migration of extravillous trophoblasts.

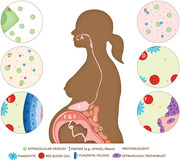

## 1 Introduction

Fetal growth restriction (FGR)—a pathological condition in which the fetus fails to reach its genetically determined growth potential—affects nearly 10% of all pregnancies globally.^[^ ^1^, ^2^ ^]^ It has both short‐ and long‐term consequences for the offspring, including a higher incidence of stillbirth^[^ ^3^ ^]^ neonatal morbidity^[^ ^4^ ^]^ and the risk of acquiring non‐communicable diseases (e.g., cardiovascular and metabolic disease) in adulthood.^[^ ^5^, ^6^ ^]^ Currently, there are limited treatment options, other than iatrogenic premature delivery, which exacerbates poor health outcomes.^[^ ^7^ ^]^

FGR is associated with sub‐optimal development and/or function of the placenta. In normal placental development, precursor mononuclear cytotrophoblast cells proliferate and differentiate into one of two subtypes; extravillous trophoblast, which anchor the placenta within the maternal uterus, or syncytiotrophoblast (syncytium), which is responsible for nutrient and gas exchange. Fetal nutrient demands increase as gestation progresses, requiring the placenta to increase its surface area and transfer capacity.^[^ ^8^ ^]^ Though transcriptionally active, syncytial nuclei are postmitotic thus expansion of the syncytial surface occurs through proliferation, differentiation, and fusion of the underlying cytotrophoblast. Our previous work has established that maternal hormones and growth factors regulate the rate of these processes.^[^ ^9^ ^]^ However, there is little mechanistic information on how other signals derived from the maternal circulation, including those from maternal diet, regulate placental growth, and function. A maternal diet rich in fruit and vegetables (F&V) is associated with good fetal growth,^[^ ^10^, ^11^, ^12^, ^13^, ^14^ ^]^ however the molecular basis of the relationship is not fully understood as it cannot been explained by known macro‐/micro‐nutrients^[^ ^12^, ^15^ ^]^ or socioeconomic confounders.^[^ ^16^ ^]^ Studies of other organ systems have demonstrated that fruit and vegetables release extracellular vesicles (EVs)—cell‐derived lipid‐bound vesicles containing proteins and RNA—that exert beneficial effects in mammals, e.g., ginger‐derived EVs protect mice from alcohol‐induced liver injury^[^ ^17^ ^]^ and grape “EV‐like” nanoparticles protect mice from dextran sulfate sodium‐induced colitis.^[^ ^18^ ^]^ Other work has demonstrated that animal‐based dietary products can traverse the gut barrier and release EVs into the circulation and that these EVs can modulate gene/protein expression and biological functions in both the gut and distal organs.^[^ ^19^, ^20^ ^]^ Moreover, recent work in mice has shown that EVs from ingested bovine milk can accumulate in placenta and promote embryo survival.^[^ ^21^ ^]^

Our previous work has shown that non‐dietary EVs within maternal circulation can enter the human placenta and exert functional effects.^[^ ^22^ ^]^ Therefore here, we investigated if, via actions on placenta, EVs could be responsible for the beneficial effects of maternal F&V intake on fetal growth. We chose EVs derived from watermelon (*Citrullus lanatus*) as the exemplar for these proof‐of‐principle studies since in humans, components of watermelon (WM) EVs have been detected in the circulation following consumption of watermelon juice,^[^ ^23^ ^]^ and there is a trend for reduced incidence of small‐for‐gestational‐age infants when mothers eat watermelon during pregnancy.^[^ ^24^ ^]^

We show that EVs from watermelon fruit contain miRNA and proteins which, according to bioinformatic analyses, have the potential to affect mammalian cell function. We confirm this prediction by demonstrating that watermelon EVs are internalized by human intestinal cells to alter the intestinal cell secretome and that this altered secretome promotes two key aspects of human placental cell behavior, trophoblast migration, and fusion.

## 2 Results

### 2.1 Watermelon Fruit Contain Extracellular Vesicles that Influence Watermelon and Mammalian Cell Function

**Figure 1:**
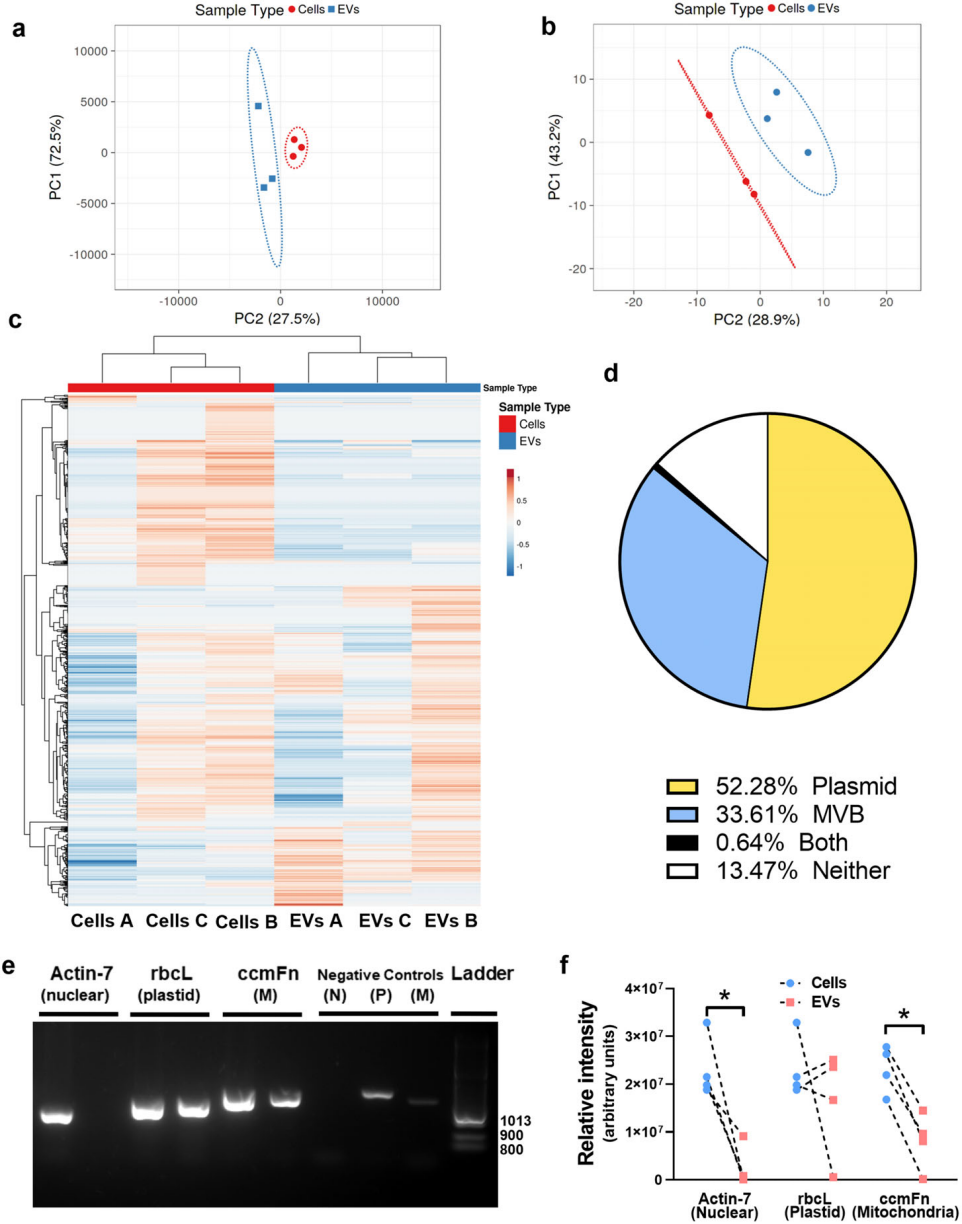
Watermelon extracellular vesicles contain a miRNA, protein, and DNA profile which differs from that of watermelon cells and predicts a dual plastid and mitochondrial site of biogenesis. a) The levels of miRNAs in watermelon mesocarp cells and mesocarp‐derived‐EVs were determined using the rice miFinder qPCR array (*n* = 3) and principal components analysis of miRNAs in mesocarp cells and EVs was performed on log10 ∆∆Ct values. b and c) Watermelon EV proteins were profiled using mass spectrometry and (b) analyzed using principal components analysis or (c) hierarchical clustering. Data are log10 transformed (relative abundance); *n* = 3. Hierarchical clustering was performed with correlation distance and complete linkage for columns and average distance for rows. d) GO enrichment analysis was performed to determine potential origin of EV proteins (Bonferroni *p*‐value correction; CELLO program^[^ ^31^ ^]^) and complemented with published subcellular localization.^[^ ^32^ ^]^ The chart shows the percentage of total watermelon EV proteins that are predicted and/or reported to reside within plastids or multivesicular bodies (MVB), both or neither organelle. e) DNA was extracted from watermelon cells and EVs and analyzed by PCR to determine cellular localization of isolated DNA using primers for DNA of nuclear (N; actin) plastid (P; rbcL) and mitochondria (M; ccmFn) origin. f) Quantification of PCR data shows that EVs have lower levels of nuclear and mitochondrial DNA than cells but appear to have similar levels of plastid DNA. Data are shown as median (Mann–Whitney *U* test; * = *p* < 0.05).

Juice derived from *C. lanatus* (watermelon) fruit contained particles of a similar size (Figure S1a,b, Supporting Information) and morphology (Figure S1c, Supporting Information) to ALIX^+ve^/CD63^+ve^/CANX^–ve^ EVs isolated from human intestinal epithelial (Caco‐2) cells (Figure S1d, Supporting Information). Watermelon EV (WMEV) miRNA (**Figure** **1**a; Table S1, Supporting Information) and protein (Figure 1b,c; Table S1, Supporting Information) profiles differed from those of watermelon fruit cells. Further, WMEVs were not contaminated with proteins usually found in other cellular compartments, miRNA presence was not dependent on sequence and the levels of enriched miRNAs and their predicted protein targets were inversely correlated (Figure S1e, Supporting Information), suggesting active cargo sorting, consistent with the findings of other studies.^[^ ^25^ ^]^ Currently, there are no reliable markers for plant‐derived EVs,^[^ ^26^ ^]^ however comparison (Table S2, Supporting Information) of the WMEV proteome with published data on EV‐like particles isolated from other plant species^[^ ^27^, ^28^, ^29^, ^30^ ^]^ revealed common proteins, including S adenosylhomocysteine, that are postulated to be candidate markers of plant EVs.^[^ ^26^ ^]^ Moreover, proteomic data suggest that as well as multivesicular bodies (MVBs), which are the source of the exosome EV subtype in animals, plant EVs originate from additional organelles; analyses of the protein (Figures 1d) and DNA (Figure 1e,f) implicate plastids.

Interestingly, 23% of the proteins detected in WMEVs have a human homolog with a sequence identity >50% and coverage >70%. Functional enrichment analysis (Figure S2a, Supporting Information) suggests such proteins are involved in human metabolism, gene expression/protein production, and cell death. Similar analysis of the predicted human targets of WMEV miRNAs (Figure S2b, Supporting Information) also revealed a role in cell death in addition to other roles in development, cell cytoskeletal dynamics/motility, and proliferation. Together these data suggest that mammalian cell function, including viability, may be altered by WMEV cargo.

### 2.2 Watermelon Extracellular Vesicles Are Internalized by Human Intestinal Cells and Alter Both Their Function and Secretome

**Figure 2:**
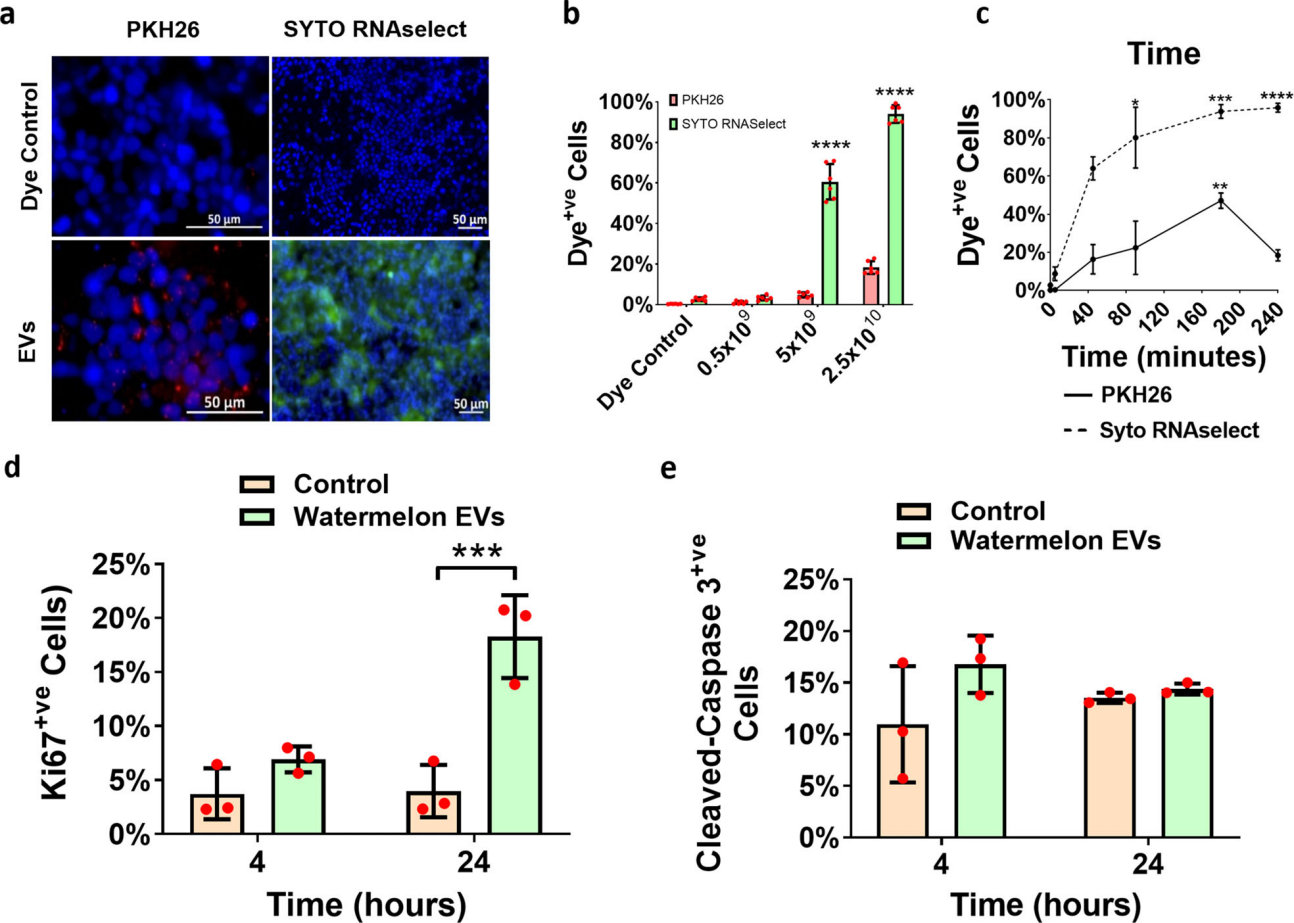
Watermelon extracellular vesicles and their RNA cargo are internalized into human intestinal epithelial cells and functionally active. a) Watermelon extracellular vesicles (WMEVs) were fluorescently labeled with the lipophilic dye PKH26 (WMEV^PKH26^; membrane labeling; Red) or the RNA‐binding dye SYTO RNAselect (WMEV^RNAselect^; RNA cargo labeling; green) and dye control or 2.5 × 10^10^ mL^−1^ labeled EVs were applied to differentiated Caco‐2 cells for 4 h. Concentration b) and time c) dependency of WMEV uptake was assessed by flow cytometry of trypsinized Caco‐2 cells treated with 5 × 10^8^–2.5 × 10^10^ mL^−1^ EVs for 4 h or 2.5 × 10^10^ mL^−1^ EVs for 5–240 min respectively. Data are expressed as percentage of PKH26^+ve^ or RNASelect^+ve^ cells and presented as median ± interquartile range. *n* = 5–6, Kruskal–Wallis test with Dunn's multiple comparisons test (comparison to 0 min), * = *p* < 0.05, **** = *p* < 0.0001. The impact of WMEVs on Caco‐2 cell proliferation d) and apoptosis e) was assessed by immunocytochemical analysis of differentiated Caco‐2 cells incubated apically with 2.5 × 10^10^ mL^−1^ unlabeled WMEVs for 240 min then fixed or switched to fresh medium and cultured for a further 20 h prior to fixation, using primary antibody against Ki67or cleaved caspase, respectively.

WMEV (fluorescently labelled to track membrane (WMEV^PKH26^) or RNA cargo (WMEV^RNAselect^)) are internalized (**Figure** **2**a) into Caco‐2 intestinal epithelial cells (differentiated to form a tight barrier that models intestine) in a concentration‐ and time‐dependent manner (Figure 2b,c.) Analysis of WMEV^PKH26^ localization in conjunction with endosomal markers (Figure S3a,b, Supporting Information) and following pre‐incubation of Caco‐2 cells with inhibitors (Figure S3c, Supporting Information), suggests that like mammalian EVs, uptake is predominantly via clathrin‐mediated endocytosis.

Analysis of the impact of WMEVs on Caco‐2 cell function, guided by in‐silico analyses (Figure S2c,d, Supporting Information), revealed that proliferation (Figures 2d), but not apoptosis (Figures 2e), was significantly increased 24 h after incubation (4 h) with WMEV demonstrating that as predicted, WMEVs are functionally active in mammalian cells.

**Figure 3:**
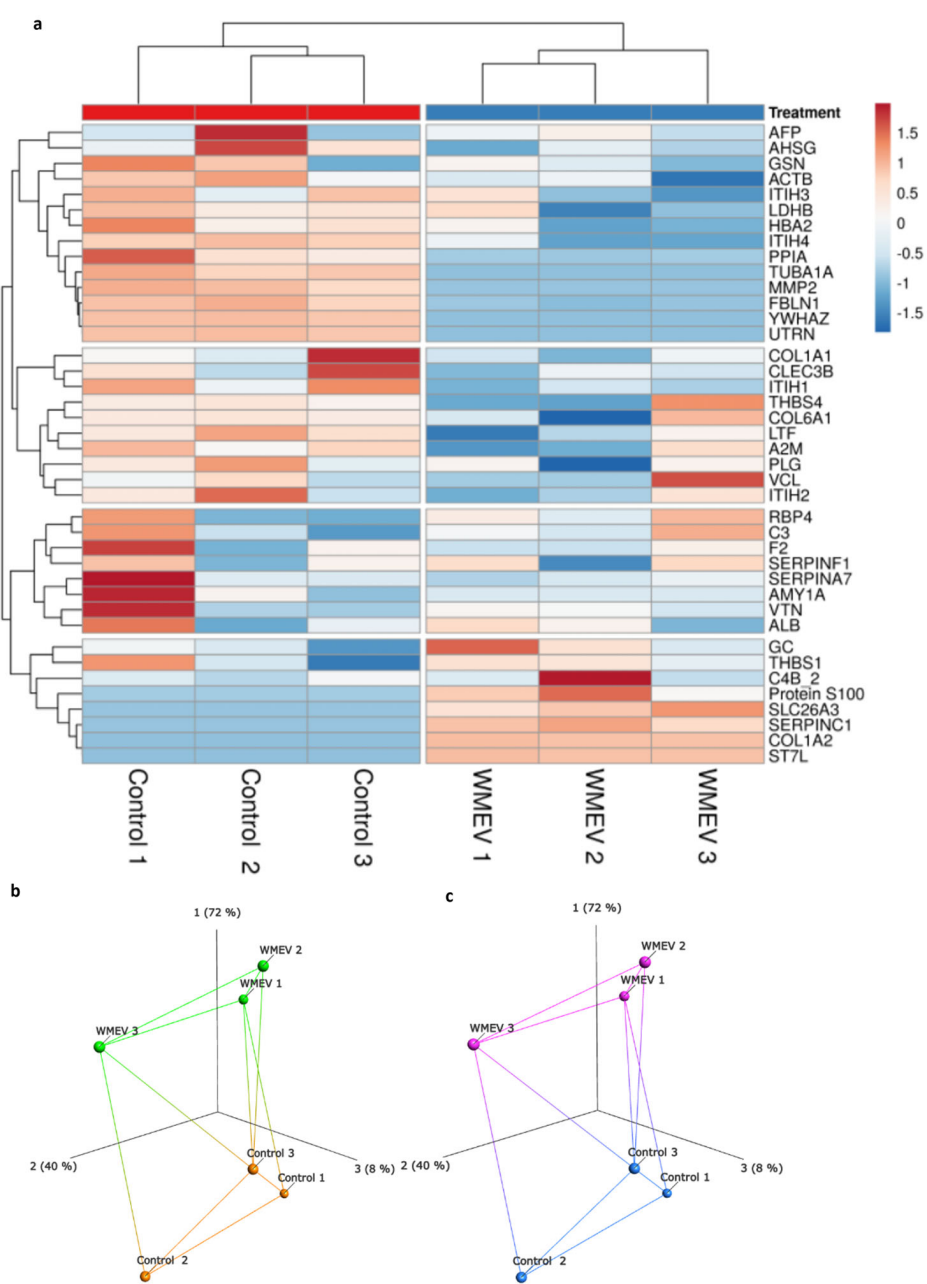
Incubation with watermelon extracellular vesicles alters the basal secretome of Caco‐2 cells. a) Basal medium was collected from Caco‐2 cells incubated for 240 min in the presence or absence of 2.5 × 10^10^ watermelon extracellular vesicles (WMEV) mL^−1^ in the apical medium compartment (*n* = 3). The profile of proteins in basal medium was determined by mass spectrometry; 40 proteins were detected. Data were analyzed using undirected hierarchical clustering analysis using Euclidean clustering and complete linkage. Protein profiles were then compared by isomap analysis with b) labeling of treatment group or c) unbiased labeling using k‐means clustering (two groups detected using an elbow plot).

We then investigated the effect of WMEV on the treated Caco‐2 cell secretome, focussing on the protein content of the secretome as in vivo transfer of ingested, EV‐derived miRNA across the gut to the circulation is controversial.^[^ ^33^ ^]^ Proteomic analyses of all 40 proteins detected (**Table** **1**, Figures **3** and S4, Supporting Information) indicated that while no watermelon‐specific proteins were present in the secretome, the profile of human proteins is altered following exposure to WMEV, confirmed by hierarchical clustering and isomap analysis (Figure 3), with the abundance of 5 and 11 proteins up‐ and down‐regulated respectively. Ingenuity pathway analysis of each of the four clusters depicted in Figure 3 revealed roles associated with apoptosis, cell movement, microtubule dynamics, lipid synthesis and molecule transport; all are pertinent to the regulation of placental function, though other distal organs are likely impacted by WMEV‐induced changes to the intestinal secretome also. Furthermore, holistic (IPA and PathwAX) analysis of the entire dataset suggested that immune system‐related pathways are enriched in the altered secretome (Figure S4, Supporting Information).

**Table 1:** Incubation with watermelon extracellular vesicles alters the intestinal epithelial cell secretome

Gene name	Protein name	Caco‐2 basal media proteins watermelon EV‐exposed versus control(fold‐change; *n* = 3; mean ± standard deviation)
**S100A9**	**Protein S100‐A9**	**12.96** ± **5.70**
**SERPINC1**	**Antithrombin‐III**	**8.29** ± **1.03**
**SLC26A3**	**Solute carrier family 26 member 3**	**6.00** ± **1.26**
**COL1A2**	**Collagen type I alpha 2 chain**	**4.71** ± **0.04**
**ST7L**	**Suppression of tumorigenicity 7 like**	**4.71** ± **0.04**
**C3**	**Complement component 3**	**1.39** ± **3.18**
**F2**	**Coagulation factor II, thrombin**	**1.32** ± **2.13**
**C4B_2**	**Complement component 4B**	**1.21** ± **3.03**
**GC**	**GC, vitamin D binding protein**	**1.19** ± **0.04**
**VTN**	**Vitronectin**	**1.08** ± **1.70**
**ALB**	**Albumin**	**1.03** ± **2.19**
**AFP**	**Alpha fetoprotein**	**1.01** ± **0.33**
**COL6A1**	**Collagen type VI alpha 1 chain**	**1.00** ± **1.99**
**VCL**	**Vinculin**	**–1.01** ± **4.73**
**SERPINA7**	**Serpin family A member 7**	**–1.05** ± **2.22**
**ITIH2**	**Inter‐alpha‐trypsin inhibitor heavy chain 2**	**–1.20** ± **2.57**
**SERPINF1**	**Serpin family F member 1**	**–1.23** ± **1.40**
**THBS1**	**Thrombospondin 1**	**–1.23** ± **1.86**
**RBP4**	**Retinol binding protein 4**	**–1.27** ± **1.90**
**CLEC3B**	**C‐type lectin domain family 3 member B**	**–1.28** ± **2.37**
**HBA2**	**Hemoglobin subunit alpha 2**	**–1.33** ± **0.10**
**FBLN1**	**Fibulin 1**	**–1.34** ± **0.04**
**PLG**	**Plasminogen**	**–1.35** ± **2.67**
**THBS4**	**Thrombospondin 4**	**–1.36** ± **1.46**
**A2M**	**Alpha‐2‐macroglobulin**	**–1.40** ± **0.39**
**PPIA**	**Peptidylprolyl isomerase A**	**–1.44** ± **0.17**
**COL1A1**	**Collagen type I alpha 1 chain**	**–1.46** ± **0.21**
**AHSG**	**Alpha 2‐HS glycoprotein**	**–1.50** ± **0.08**
**ACTB**	**Actin beta**	**–1.60** ± **0.34**
**ITIH3**	**Inter‐alpha‐trypsin inhibitor heavy chain 3**	**–1.70** ± **0.75**
**GSN**	**Gelsolin**	**–1.73** ± **1.68**
**LDHB**	**Lactate dehydrogenase B**	**–1.90** ± **0.84**
**ITIH4**	**Inter‐alpha‐trypsin inhibitor heavy chain 4**	**–1.91** ± **0.53**
**AMY1A**	**Amylase, alpha 1C (salivary)**	**–1.91** ± **2.05**
**LTF**	**Lactotransferrin**	**–2.13** ± **1.06**
**ITIH1**	**Inter‐alpha‐trypsin inhibitor heavy chain 1**	**–3.22** ± **1.76**
**YWHAZ**	**14‐3‐3ζ**	**–6.31** ± **0.13**
**UTRN**	**Utrophin**	**–6.31** ± **0.13**
**TUBA1A**	**Tubulin alpha 1a**	**–6.98** ± **0.67**
**MMP2**	**Matrix metallopeptidase 2**	**–7.06** ± **0.80**

Proteins from basal medium of Caco‐2 cells treated apically for 240 min with watermelon EVs or dye control were analyzed using mass spectrometry. Proteins detected in only one biological repeat were excluded. Data are presented as mean +/‐ standard deviation (*n* = 3). Forty proteins were identified in total; red shading indicated proteins that were significantly increased by >1.5 fold and gray indicated proteins that were decreased by >1.5 fold in basal media following exposure to watermelon EVs.

### 2.3 Secretome of WMEV‐Treated Intestinal Epithelial Cells Modulates Human Placental Proteome and Function

**Figure 4:**
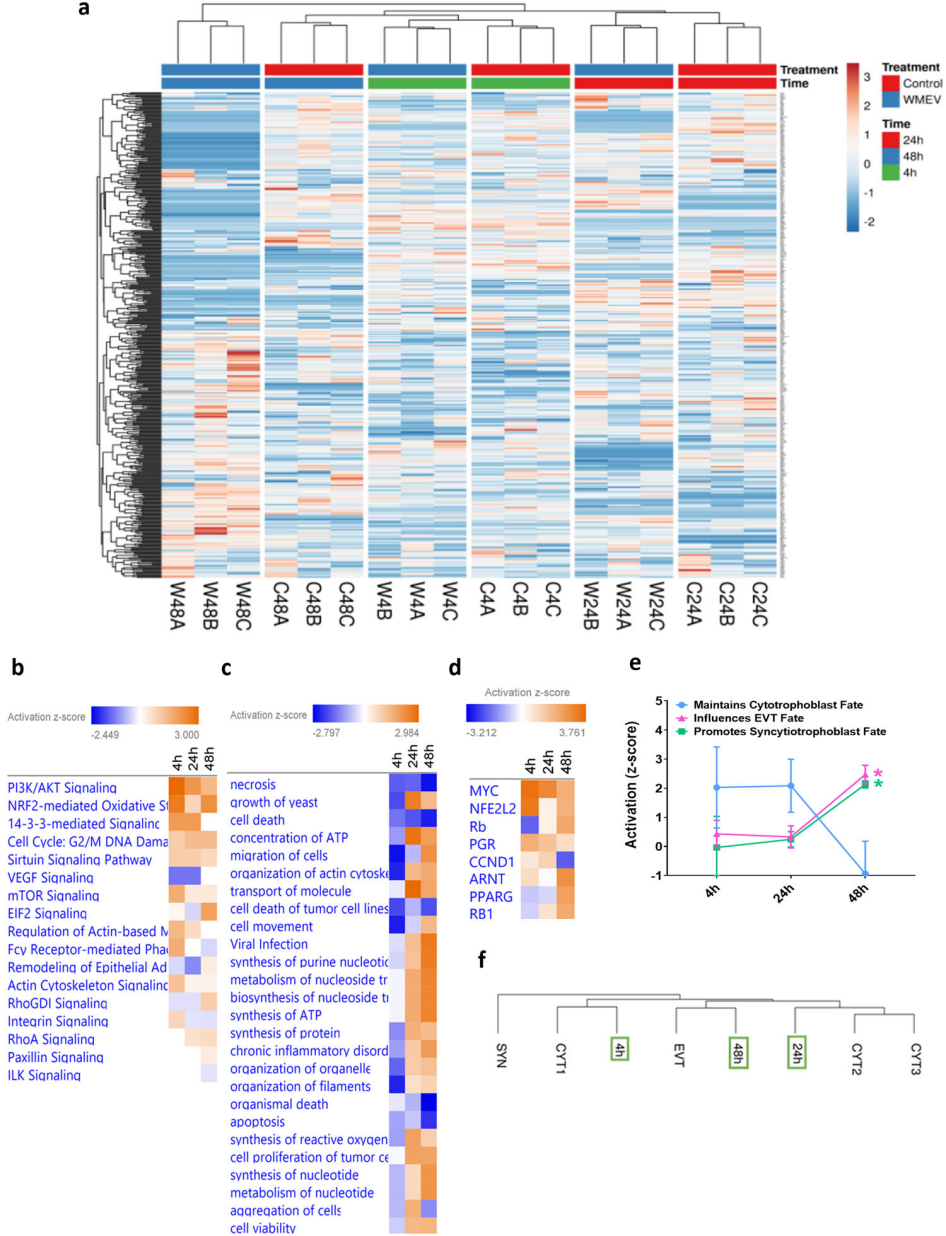
Watermelon extracellular vesicles alters the impact of intestinal epithelial cell secretome on human placental trophoblast proteome. Basal conditioned medium was obtained from untreated (control) Caco‐2 cells or Caco‐2 cells cultured apically with 2.5 × 10^10^ mL^−1^ watermelon extracellular vesicles (WMEV) for 4 h (*n* = 6 pooled), and applied to BeWo cells for 4, 24, or 48 h. Mass spectrometry was used to characterize the proteome of these BeWo cells. a) Hierarchical clustering analysis (Euclidean clustering, complete linkage) of all proteins detected in lysates from BeWo cells incubated with basal medium from Caco‐2 control‐ (C) or WMEV (W) treated‐ cells (*n* = 3, labeled A–C) at 4, 24, or 48 h. BeWo cell proteomes (all 448 proteins) were subjected to b) Ingenuity pathway, c) functional, and d) upstream regulator enrichment analyses to predict potential impact on placental function. Color coding shows Z‐score value for predicted activation (>0) or inactivation (<0) of pathway activity. e) The identified transcription factors with known roles in trophoblast biology were classified by their influence on trophoblast differentiation in the following categories: “Maintains cytotrophoblast fate” (blue); “Promotes EVT fate” (red), and “Promotes syncytiotrophoblast fate” (green). Data are presented as mean ± standard deviation. Two‐Way ANOVA with Sidak's multiple comparisons test, * = *p* < 0.05. e) The proteome of BeWo cells exposed to WMEV‐induced intestinal secretome (fold‐change from time‐matched control) were compared with the published transcriptome of primary trophoblast subtypes.^[^ ^34^ ^]^ Syncytiotrophoblast (SYN), extravillous trophoblast (EVT), undifferentiated primary cytotrophoblast (CYT1), or differentiating primary cytotrophoblast (CYT2 and CYT3). BeWo cells treated for 4 h cluster with undifferentiated cytotrophoblast (CYT); 24 h‐treated cells cluster with differentiating (syncytializing) cytotrophoblast (CYT2, CYT3); 48 h‐treated cells cluster closely with EVTs.

Next, we demonstrated that the placental trophoblast cell line, BeWo, internalizes Caco‐2‐secreted EVs by clathrin‐mediated endocytosis into late endosomes/lysosomes (LAMP1^+ve^; Figure S5, Supporting Information), suggesting that intestinal‐placental communication can occur. Indeed, treating BeWo cells with the secretome of Caco‐2 cells exposed to WMEVs induced temporal changes in the BeWo proteome (**Figures** **4**a).

Functional enrichment analysis of altered proteins (Figure 4b,c), together with analysis of the upstream transcription factors predicted to be responsible for such changes (Figure 4d,e) and comparison to single cell RNA‐seq data from human placentas^[^ ^34^ ^]^ (Figure 4f), suggests that prolonged exposure (48 h) of placental cells to the secretome of WMEV‐treated Caco‐2 cells influences trophoblast differentiation along syncytial and invasive pathways.

**Figure 5:**
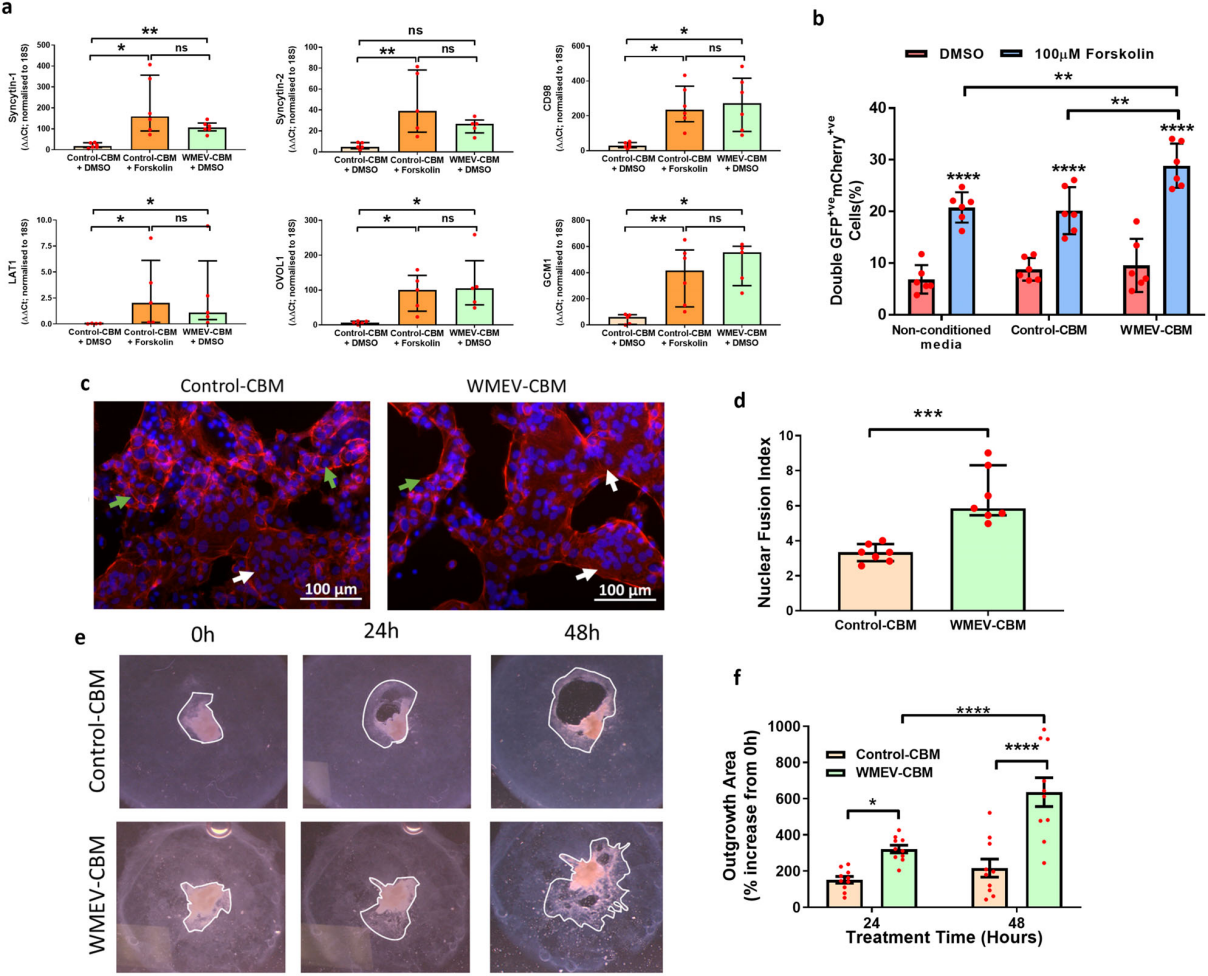
BeWo cells and primary human cytotrophoblast function is enhanced following exposure to secretome from watermelon extracellular vesicle‐treated intestinal cells. a) BeWo cells were incubated for 48 h in the secretome from untreated (Control‐CBM) Caco‐2 cells supplemented with either 0.5% DMSO (carrier control) or forskolin (100 µM) or in the secretome from Caco‐2 cells apically exposed to watermelon extracellular vesicles (WMEV‐CBM). The expression levels of six genes involved in syncytialization were analyzed using qPCR and normalized to 18S rRNA. b) 50:50 cultures (*n* = 6) of GFP^+ve^BeWo:mCherry^+ve^BeWo cells were incubated for 48 h with non‐conditioned medium, control CBM, or WMEV‐CBM in the absence or presence of forskolin for 48 h before determining the percentage of fused (double positive) cells by flow cytometry. c) Freshly isolated primary human cytotrophoblasts were incubated with Control‐CBM or WMEV‐CBM for 48 h then fixed and stained for phalloidin (red) and DAPI (Blue). d) Fusion index (average number of nuclei/cell) was calculated from analysis of images using Image J. All data are presented as median ± interquartile range. ns = not significant, * = *p* < 0.05, ** = *p* < 0.01, *** = *p* < 0.001, **** = *p* < 0.0001. Explants of first trimester human placenta were plated on collagen and cultured for 24 h prior to exposure to the secretome of control (control‐CBM) or WMEV‐exposed Caco‐2 cells (WMEV‐CBM) for a further 48 h. e) The outgrowth/migration of extravillous trophoblasts was quantified using ImageJ from f) light‐field images. Data are presented as mean ± standard deviation; *n* = 3; Two‐way ANOVA * = *p* < 0.01; **** = *p* < 0.0001.

We tested this hypothesis firstly by assessing the effect of the WMEV‐treated Caco‐2 cell secretome on trophoblast syncytialization, a key process in expanding placental surface area and capacity for maternal‐fetal nutrient transfer. The secretome does indeed promote syncytialization, of both BeWo cells and primary human cytotrophoblast, with upregulation of key genes (**Figure** **5**a) and enhanced fusion of BeWo (Figure 5b) and primary trophoblast cells (Figures 5c,d).

Furthermore, the EV‐induced Caco‐2 cell secretome also enhanced the outgrowth (invasion) of extravillous trophoblast (EVT) from human first trimester placental villous explants by 3‐fold compared to the secretome from untreated cells (Figure 5e,f).

## 3 Discussion

Here we present the first data on the isolation and characterization of EVs from watermelon, and their cross‐species communication with human cells. We show that watermelon EVs are actively taken up into gut epithelial cells by clathrin‐mediated endocytosis to modify the basal secretome, which is representative of the post‐absorptive human circulation and that this altered secretome enhances two important processes for efficient placental function—trophoblast invasion and syncytialization. These data provide a mechanistic explanation for the beneficial effect of dietary F&V on fetal growth and reveal a novel route for improving placental function and pregnancy outcome.

Watermelon EVs are of a size and morphology reminiscent of mammalian EVs/exosomes,^[^ ^35^ ^]^ and carry a diverse, selectively packaged, protein, and miRNA cargo suggesting that they also contribute to organismal control mechanisms. Our bioinformatic analyses predict a role in the fruit's metabolism and development/ripening and identifies a core set of proteins that could be used to facilitate EV identification in plants, fulfilling a recognized need^[^ ^36^ ^]^ for standardization amongst the field in order to better understanding their biogenesis, distribution, and functions in plants.

Importantly, our data also predicted, and experimentally confirmed, that WMEVs can influence the function of human cells. We have shown that watermelon EVs are actively taken up by human intestinal (Caco‐2) cells in vitro through clathrin‐mediated endocytosis, as previously shown for Caco‐2 cell import of bovine milk EVs.^[^ ^37^ ^]^ Although our EV preparations were not subjected to mimics of gastric or intestinal juice, which may affect their charge and therefore uptake,^[^ ^38^ ^]^ these data are in agreement with in vivo studies demonstrating that EVs isolated from a range of F&V and other plants are internalized by cells of the murine^[^ ^18^, ^38^, ^39^ ^]^ and porcine^[^ ^39^ ^]^ gut. We also found that, as predicted by in silico analysis of WMEV miRNAs and protein, proliferation of Caco‐2 cells was enhanced by exposure to WMEV, though cell survival was not affected. Others have reported that EVs from other plant species, e.g., ginger, influence this aspect of cell function only in the presence of an apoptosis‐inducing insult.^[^ ^40^ ^]^

Currently, it is not known if intact plant EVs or merely their contents, perhaps re‐packaged into host EVs, are transferred across the gut epithelial barrier and indeed, the transfer and regulatory role of miRNA across kingdoms is controversial. Nonetheless, the potential for dietary‐derived EVs to influence the function of cells and tissues distal to the gut by affecting the gut epithelium, immune cells and/or microbiome is an area of intense research.^[^ ^33^ ^]^ Plant EVs are internalized by intestinal macrophages^[^ ^38^, ^41^ ^]^ and we have previously demonstrated that the placenta internalizes macrophage EVs^[^ ^22^ ^]^ suggesting one potential route for dietary EV cargo to reach the placenta. Additionally, or alternatively, EVs may indirectly influence distal cells by altering molecules released from the gut. Indeed, we have found that the protein content of the intestinal cell secretome is altered by exposure to watermelon EVs. These changes were apparent after treating only once with a dose of watermelon EVs consistent with the amount present in a single serving of juice or fruit, and although further work to mimic the effects of repeated eating is required, our data show that the placental proteome is affected by the altered intestinal secretome, providing proof‐of‐principle for dietary EV‐induced modification of placental function.

In‐silico analysis of the trophoblast proteome suggests the nutrient‐sensitive mammalian target of rapamycin (mTOR) signaling pathway is activated in response to the secretome from gut exposed to WMEV, presenting a mechanism for the gut to convey information on nutrient availability to the placenta and influence key aspects of placental development and function, e.g., survival, migration, and transport. These predictions are supported by our demonstration that this secretome increased EVT migration from explants of human first trimester placenta, a key step in spiral artery remodeling and delivery of nutrient‐rich blood to the placenta. Whether this is due to a direct effect on cell mobility and/or differentiation of cytotrophoblasts to a migratory phenotype remains to be determined.

In addition to effects on trophoblast invasion, we also show modulation of trophoblast syncytialization. Gene ontology analysis of the changes to the trophoblast proteome in response to the EV‐induced gut secretome, in conjunction with the upregulation in fusion‐related gene expression, and enhancement of BeWo and primary cytotrophoblast syncytialization, suggests that WMEVs also improve trophoblast syncytialization, a key process in developing and maintaining nutrient transfer capacity. As deficits in syncytialization and transport of nutrients into and across the placenta are characteristics of the placental insufficiency associated with FGR, WMEV‐mediated modification of these processes could provide a much‐needed strategy for the treatment or prevention of FGR that overcomes many of the issues associated with the development and use of new drugs in pregnancy.^[^ ^42^ ^]^

Work from other groups supports the potential for plant/fruit components as therapeutics in pregnancy. Extracts from the leaves of lowbush blueberry have been shown to affect the migration of a trophoblast cell line,^[^ ^43^ ^]^ likely due to the presence of the phenolic compound chlorogenic acid. In addition, the pomegranate polyphenol, punicalagin, promotes syncytialization of primary human cytotrophoblast as well as protecting the syncytium from hypoxia‐induced cell death.^[^ ^44^, ^45^ ^]^

Finally, proteomic analysis of the EV‐induced gut secretome indicates the potential for EVs from F&V to regulate metabolism and reducing inflammation, both within the intestine, as documented by studies of EVs from other plant species,^[^ ^18^, ^38^, ^41^, ^46^, ^47^ ^]^ and systemically. If substantiated, such findings will be of interest in relation to the development of therapeutic strategies for metabolic complications of pregnancy, such as diabetes and obesity, and/or preeclampsia, a pregnancy complication rooted in placental dysfunction that is characterized by high levels of inflammation. Interestingly, adverse outcomes in pregnancies complicated by diabetes and the incidence of preeclampsia are reduced in mothers whose diet is rich in F&V.^[^ ^13^, ^48^ ^]^

It is difficult to identify placental developmental/functional anomalies with precision, necessitating treatment of women at risk of developing complications such as FGR; some of these women may transpire to have normal pregnancies, meaning that any potential treatment must have minimal, ideally zero, risk. The “two patient problem” of the mother‐fetal dyad, exposed by the use of thalidomide during pregnancy, curtailed pharma interest in pregnancy disease; contemporary studies have tried to re‐purpose drugs developed for other conditions that are thought “safe” in pregnancy. However, even this approach is beset by stakeholder apprehension, which has led to poor compliance in some studies^[^ ^49^, ^50^ ^]^ and premature cessation of others.^[^ ^51^ ^]^ Against this backdrop, our data suggesting that watermelon‐derived EVs have the potential to enhance placental growth and activity supports the need for further investigation of the potential for plant‐derived EVs, which are rapidly gaining traction as therapeutic agents^[^ ^19^ ^]^ and delivery systems^[^ ^52^ ^]^ for a range of pathological conditions, as a novel, natural intervention form women at risk of FGR. A prospective clinical study would now be justified.

## 4 Experimental Section

### Extracellular Vesicle Isolation

Watermelons (*n* = 9) were purchased from a range of local greengrocers and supermarkets. Mesocarp was removed, pulsed for 1 s in a standard kitchen blender, then sieved to generate an opaque juice. Cells and debris were removed by centrifugation at 1000 × *g* for 10 min, 2× 10,000 × *g* for 10 min; A‐621 fixed‐angle rotor at 4 °C using a Sorvall Discovery 100SE centrifuge (Sorvall, UK) set to “RCFavg.” The resulting supernatant was filtered (0.22 µm) then centrifuged at 100,000 × *g* for 90 min at 4 °C using the T‐1250 fixed‐angle rotor to pellet EVs. The harvested pellet was resuspended in phosphate buffered saline or diluent C. Caco‐2 EVs were similarly isolated from culture medium (Dulbecco's modified Eagle medium (DMEM), 2 mM non‐essential amino acids, 4 mM glutamine, 100 U L^−1^ penicillin, 100 µg L^−1^ streptomycin, and 10% (v/v) EV‐depleted fetal bovine serum (Exo‐FBS; Cambridge Bioscience Limited, UK)) harvested 72 h after seeding at 0.1 × 10^6^/3.5 cm^3^.

### Extracellular Vesicle Characterization

EV size and concentration were determined using nanoparticle tracking analysis on a NanoSight LM10 (Malvern Instruments, UK). 3 × 60 s videos per sample were taken (temperature: 25 °C; flow rate: 30 µL s^−1^; camera type: sCMOS; laser type: Blue 405 nm; camera level: 10; 20–100 particles/frame) and analyzed using the Nanosight NTA 2.3 software (Malvern Instruments, UK) with a detection threshold of 5.^[^ ^53^ ^]^ EV morphology was determined using transmission electron microscopy (JEM1400; Jeol, USA) following negative staining with 1% uranyl acetate. Images were captured at 120 kV using an 1k CCD camera (Advanced Microscopy Techniques, Corp., USA).

Protein harvested from Caco‐2 cells and EVs (30 ng) was resolved by electrophoresis in reducing conditions, transferred to nitrocellulose membranes then probed with primary antibodies β‐catenin, (0.1 µg mL^−1^; A1978; Sigma‐Aldrich, UK), ALIX (ALG‐2‐interacting protein; final concentration 1 µg mL^−1^; ab24335; Abcam, UK), CD63 (0.5 µg mL^−1^; ab199921; Abcam), or CANX (Calnexin; 1 µg mL^−1^; A303‐695A; Bethyl Laboratories, USA) overnight at 4 °C. Secondary antibodies (1 h, room temperature) were IRDye 800CW conjugated anti‐rabbit (926‐32213) or mouse (926‐68073) IgG (0.2 µg mL^−1^; Li‐Cor Biosciences, UK Ltd., UK). Blots were imaged on the Li‐Cor Odyssey Sa (Li‐Cor Biosciences, UK Ltd., UK).

### Cell Culture

Caco‐2 cells (ECACC #86010202) were routinely maintained at <70% confluence in DMEM with the addition of 2 mM non‐essential amino acids, 4 mM glutamine, 100 U L^−1^ penicillin, 100 µg L^−1^ streptomycin, and 10% (v/v) FBS at 37 °C and 5% CO_2_. Differentiation was achieved by growing cells on polycarbonate transwells (0.4 µm pores; Corning, UK) to 100% confluency and then culturing for a further 21 days.

BeWo cells (ECACC #86082803) were cultured in a 50:50 mix of DMEM and Ham's F12 with 4 mM glutamine, 100 U L^−1^ penicillin, 100 µg L^−1^ streptomycin, and 10% FBS.

### Extracellular Vesicle Labeling

Isolated EVs were labeled with PKH26 (red; Sigma‐Aldrich, UK) or SYTO RNAselect (green; Thermo Fisher Scientific UK Ltd., UK), to indicate membrane and RNA cargo respectively then passed through 7K molecular weight cut‐off Desalting Columns; (Thermo Fisher Scientific UK Ltd., UK). Dye controls were created using the same procedures but in the absence of EVs.

### Dosage Information/Dosage Regimen

Caco‐2 cells were treated with 2.5 × 10^10^ EVs mL^−1^, to reflect the EV yield from watermelon juice (1.85 × 10^10^ ± 1.63 × 10^10^ particles mL^−1^ juice), for 4 h. Assessment of Caco‐2 cell apoptosis was used to determine if the EV treatment regimen detrimentally affected viability.

### Extracellular Vesicle Uptake

WMEVs diluted in Caco‐2 culture medium containing 10% Exo‐FBS, or an equal volume of dye control, were applied to the apical media compartment of Caco‐2 cells, differentiated in transwells for 21 days post‐confluence and EVs, at 2.5 × 10^10^ particles mL^−1^ for 240 min. In some experiments, cells were preincubated with uptake inhibitors (all Sigma‐Aldrich, UK) or vehicle control for 60 min prior to addition of labeled EVs; chlorpromazine (20–40 mg mL^−1^), dynosore hydrate (12 µM), filipin III (2 µg mL^−1^), methyl‐β‐cyclodextrin (10 mM), amiloride (400 µM), and cytochalasin D (2 µg mL^−1^). Cells were detached from membranes using 0.5 g L^−1^ trypsin then fixed for analysis of EV uptake (percentage of cells with florescence above labeling control levels) by flow cytometry using a custom‐built BD LSRFortessa (BD Biosciences, UK) and Flowing Software 2.0 (http://flowingsoftware.btk.fi/). BeWo cell uptake of Caco‐2 derived EVs was similarly assessed.

Internalized EVs were also visualized by fluorescence microscopy, and their co‐localization with cellular compartments determined using primary antibodies from Abcam, UK (rabbit anti‐CD63; 10 µg mL^−1^) or BD Transduction laboratories, USA (mouse anti‐clathrin, ‐β‐catenin, ‐zona occludins‐1 (ZO‐1), ‐Ras‐associated binding (Rab)‐4, ‐Rab5a, glucose‐regulating protein 78 (GRP78) (all 2.5 µg mL^−1^), ‐early endosome antigen‐1 (EEA‐1; 1.25 µg mL^−1^), ‐lysosome‐associated membrane glycoprotein‐1 (LAMP‐1; 50 ng mL^−1^)). Images were captured immediately using a Zeiss Observer (Zeiss, UK). The effect of WMEVs on Caco‐2 cell proliferation and apoptosis was determined by immunofluorescence cytochemistry using rabbit anti‐Ki67 and anti‐cleaved caspase 3 respectively.

### miRNA Analysis

RNA was isolated from watermelon EVs or mesocarp cells using the miRVana miRNA Isolation Kit (Thermo Fisher Scientific UK Ltd., UK) and Plant RNA isolation Aid (Thermo Fisher Scientific UK Ltd., UK; *n* = 3). Following lysis and prior to RNA isolation, samples were spiked with cel‐miR‐39 (Qiagen, UK) to allow for normalization of extraction efficiency. Reverse transcription was performed using the miScript Plant RT kit (Qiagen, UK). miRNAs were profiled using the rice (*Oryza sativa*) qPCR miFinder array given the high homology between the two species^[^ ^54^ ^]^ and lack of a specific, commercially available assay for watermelon. Any rice miRNA with a sequence containing >2 deviations from the published watermelon sequence^[^ ^55^ ^]^ was excluded (38 miRNAs). snoR10, snoR31, snoR5‐1a, U15, and U65‐2 qPCR array housekeeping genes were used alongside the internal reverse transcription control and cel‐miR‐39 for normalization.

### Proteomics

Protein harvested (in radioimmunoprecipitation assay (RIPA) buffer; Sigma‐Aldrich, UK) from cells (*n* = 3) or EVs (600 µg; (*n* = 3)) was run 5 mm into a 10% polyacrylamide gel (Bio‐Rad, USA) prior to fixation and staining using Imperial Protein Stain (Sigma‐Aldrich, UK). The excised protein band was dehydrated using acetonitrile, dried via vacuum centrifugation then proteins were reduced (10 mM dithiothreitol), alkylated (55 mM iodoacetamide) before digestion by trypsin (overnight at 37 °C). Digested samples were analyzed by liquid chromatography coupled‐mass spectrometry/mass spectrometry (LC‐MS/MS) using an UltiMate 3000 Rapid Separation LC (Dionex Corporation, USA) coupled to an Orbitrap Elite (Thermo Fisher Scientific, USA) mass spectrometer. Peptide mixtures were separated for 44 min at 300 nL min^−1^ using a 1.7 µM Ethylene Bridged Hybrid C18 analytical column (75 mm × 250 µm internal diameter; Waters, UK) and 0.1% formic acid in a gradient of 8–33% acetonitrile. Detected peptides were then selected for fragmentation automatically by data dependant analysis. The identified peptides were mapped using Mascot 2.5.1 (Matrix Science, UK), to the *C. lanatus* or *Homo sapiens* proteome. Mascot 2.5.1 was used with a fragment tolerance of 0.60 Da (monoisotopic), a parent tolerance of 5.0 parts per million (monoisotopic), fixed modifications of +57 on C (carbamidomethyl), variable modifications of +16 on M (oxidation). A maximum of one missed cleavage was permitted. Data were validated using Scaffold (Proteome Software, USA), employing the following thresholds: 95% protein identification certainty, 1 for the minimum number of unique peptides mapping to each protein, and 80% peptide identification certainty.

### Bioinformatics Analysis

Rice miRNAs were aligned with published watermelon miRNAs using the blastn mode of BLAST, with those miRNAs with <2 mismatches along the length of the miRNA (i.e., >10% deviation from the watermelon sequence) being considered homologous. T‐Coffee Multiple Sequence Alignment was used to create a phylogenetic tree of watermelon miRNA sequences. Prediction of miRNA targets was carried out using psRNAtarget^[^ ^56^ ^]^ using the “*Citrullus lanatus* (watermelon), transcript, Cucurbit Genomics Database, version 1.”^[^ ^57^ ^]^ FASTA sequences for watermelon proteins were procured from the Cucurbit Genome Database^[^ ^57^ ^]^ and were aligned with those from other plant species and humans using the blastp mode of BLAST. An alignment was considered to predict a homologue if the following criteria were met: a query cover of at least 70% with 50% identity within this region. Human homologues were compared to the Vesiclepedia collection^[^ ^58^ ^]^ of known human EV proteins using the FUNRICH V3 program.^[^ ^59^ ^]^ In order to predict the subcellular location of EV biogenesis, the known/annotated subcellular localization of watermelon EV proteins was obtained from the Cucurbit Genome Database (Feilab, 2017). The presence of motifs required for sorting into different subcellular locations was also used to predict subcellular localization using the CELLO software.^[^ ^60^ ^]^ Alignment was also performed to the proteome of watermelon chromoplasts,^[^ ^32^ ^]^ the late endosome/MVB proteome of *Arabidopsis thaliana* ^[^ ^61^ ^]^ and transcriptomics data from ripening watermelon fruit.^[^ ^62^ ^]^

Proteins and predicted miRNA targets were analyzed for pathway overrepresentation against the whole watermelon proteome using the Bonferroni test; adjusted *p*‐values of <0.05 were considered significant. This procedure was carried out using the Cucurbit Genome Database.

Ingenuity Pathway Analysis (IPA; Qiagen, UK; https://www.qiagenbioinformatics.com/products/ingenuitypathway‐analysis) was used to predict the impact of WMEVs in human cells. Functions and pathways were considered enriched using the Fisher's exact test and the Benjamini and Hochberg method of multiple comparison adjustment. Only those functions or pathways with *z*‐score >1 were considered. Hierarchical clustering analysis and principal components analysis was performed using the ClustVis R package.^[^ ^63^ ^]^ Elbow plots were created in R and isomap analysis in Qlucore Omics Explorer 3.3.

### Human Placental Tissue Collection

First trimester placental tissue (7–10 weeks gestation) was obtained following elective medical termination of pregnancy. Term placental tissue was collected following term delivery (>37 weeks gestation) of a healthy singleton infant. Informed, written, maternal consent was obtained, and the use of tissue was approved by the North West Research Ethics Committee (13/NW/0205 and 08/H1010/55(+5) respectively).

### Syncytialization Assay

Term placentas were cut into 2 cm^3^, full thickness pieces, washed, and then dissected into fragments of villous tissue which were minced and then digested using DNAse and trypsin as previously described.^[^ ^64^ ^]^ Primary cytotrophoblast were harvested using a Percoll gradient^[^ ^64^ ^]^ and 2.5 × 10^6^ cells were plated into 35 mm dishes and cultured for 24 h in 50:50 mix of DMEM and Ham's F12 with 4 mM glutamine, 100 U L^−1^ penicillin, 100 µg L^−1^ streptomycin, and 10% FBS before being transferred into neat conditioned basal medium (CBM) from watermelon EV naïve‐ or exposed‐Caco‐2 cells for a further 48 h. The effect of Caco‐2 conditioned basal medium on syncytialization, which occurred spontaneously in primary cells^[^ ^65^ ^]^ was determined by fixing cells with 4% paraformaldehyde (15 min) then staining with phalloidin 568 and 4’,6‐diamidino‐2‐phenylindole (DAPI) in order to denote cell boundaries and nuclei respectively. Fusion index was calculated using [(*N*−*S*)/*T*] × 100, where *N* was number of nuclei in syncytia, *S* the number of syncytia, and *T* the total number of nuclei as previously described.^[^ ^53^ ^]^

### Outgrowth Assay

Following collection, first trimester placental tissue was washed then villi with opaque extravillous trophoblast (EVT)‐containing tips were identified using light microscopy and excised from the placental bed. Explants were placed on Type I collagen (Corning, USA) coated plates and cultured in a 50:50 mix of DMEM and Ham's F12 with 4 mM glutamine, 100 U L^−1^ penicillin, 100 µg L^−1^ streptomycin, and 10% FBS for 48 h. Explants that had begun to outgrow were washed with phosphate buffered saline and randomly selected for treatment with neat conditioned basal medium from either watermelon EV naive or exposed Caco‐2 cells (time 0; *n* = 3). The effect on EVT migration was assessed after a further 48 h (T48) using Image J to calculate area of migrating cells in images capture at time 0 and T48 as previously described.^[^ ^66^ ^]^

### BeWo Fusion Assay

Green fluorescent protein (GFP) and mCherry‐labeled BeWo cells were generated by transfecting wild‐type cells with 1 µg mL^−1^ plasmid DNA (pEGFP‐C1 or mCherry‐histone 2B respectively; Clontech, USA) using DharmaFECT2 (Dharmacon, USA). After 24 h, transfected cells were selected using geneticin418 (G418; 0.8 mg mL^−1^) and then maintained in medium containing 0.4 mg mL^−1^ G418. 50:50 cultures of GFP^+ve^BeWo:mCherry^+ve^BeWo were cultured for 24 h then exposed to unconditioned medium or neat conditioned basal medium from watermelon EV naïve‐ or exposed‐Caco‐2 cells for a further 48 h. 100 µM forskolin (or 0.5% dimethyl sulfoxide (DMSO)) as a vehicle control was added to some wells to induce fusion. The percentage of GFP‐positive cells that were also positive for mCherry was calculated as an indication of fusion.^[^ ^67^ ^]^

### qPCR Analysis of Syncytialization Genes

100 ng RNA extracted from BeWo cells treated with 0.5% DMSO or 100 µM forskolin (as a positive control for the induction of syncytialization^[^ ^68^ ^]^) plus neat conditioned basal medium from watermelon EV naive or exposed Caco‐2 cells for 48 h was reversed transcribed using the AffinityScriot QPCR cDNA Synthesis kit (Agilent Technologies, USA). qPCR of syncytialization genes was performed using the Brilliant III Ultra‐Fast SYBR Green QPCR Master Mix (Agilent Technologies, USA) and a Mx3005p qPCR machine (Agilent Technologies, USA)+‐. Gene expression was expressed as ΔΔCt, with 18s used as the housekeeping gene.

## Conflict of Interest

The authors declare no conflict of interest.

## Author Contributions

K.A.F. and M.W. are joint senior authors. K.F. and M.W. conceptualized the study and secured funding, with input from K.T. K.F., M.W., A.D., J.M. supervised all parts of the study. B.H. provided supervision and conceptualization in methods for extracellular vesicle isolation and analysis. K.T. performed all experiments. K.T., B.H., M.W., K.F., A.D., J.M., analyzed and interpreted data. M.W., K.F., and K.T. wrote the manuscript with input from all authors.

## Supporting information

Supporting Information

## Data Availability

We have submitted all relevant data of our experiments to the EV‐TRACK knowledgebase (EV‐TRACK ID: EV210290).^[^ ^69^ ^]^
