# Supplementary material for: Watermelon‐Derived Extracellular Vesicles Influence Human Ex Vivo Placental Cell Behavior by Altering Intestinal Secretions
Source: Mol Nutr Food Res. 2022 Aug 19;66(19):2200013. doi: 10.1002/mnfr.202200013 (PMC9787345; doi:10.1002/mnfr.202200013)
Supplement: Supplementary file 1 — Supporting Information [file MNFR-66-2200013-s001.pdf]

Supplementary Information

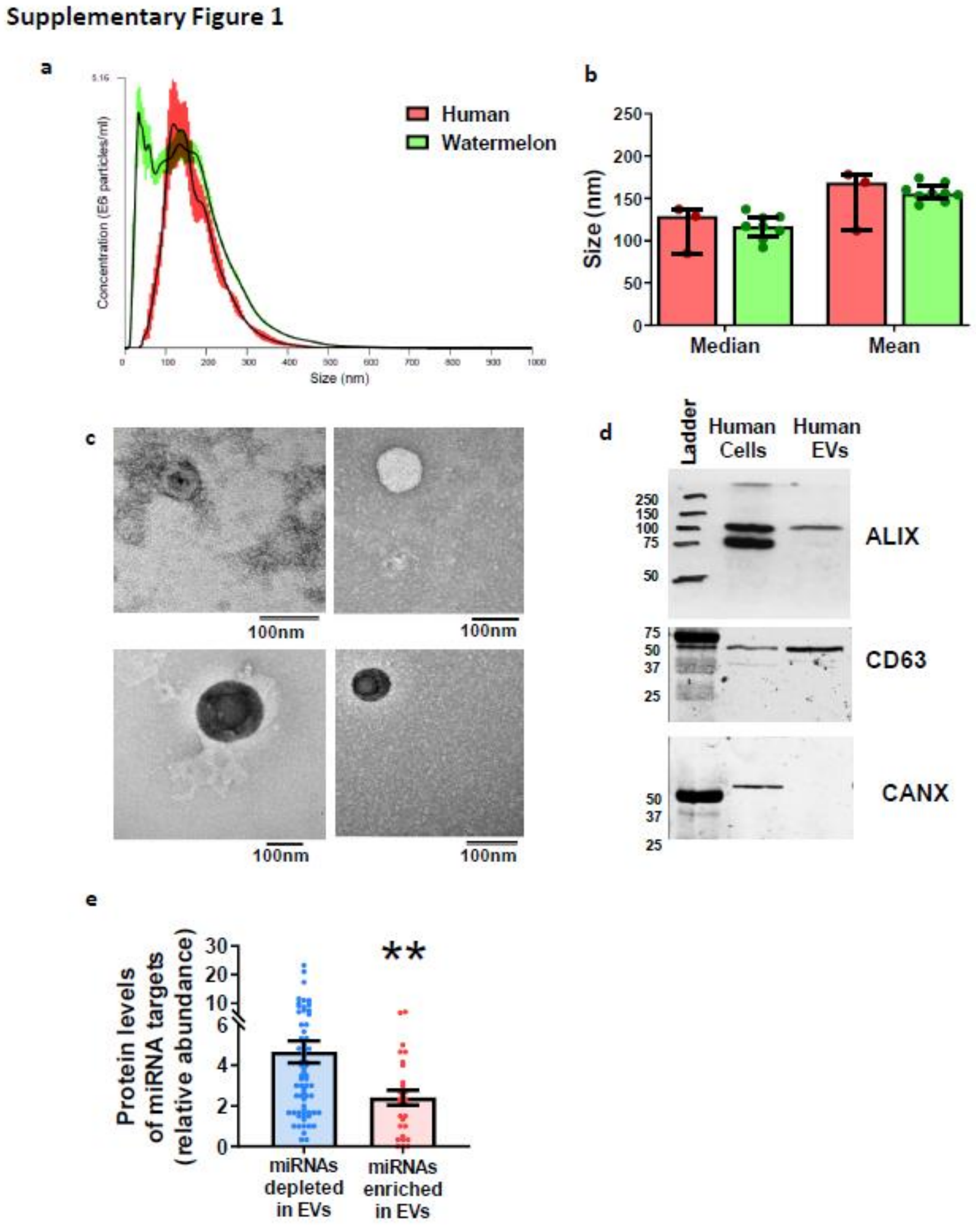

Legends

Supplementary Figure 1. Characterisation of watermelon extracellular vesicles. Extracellular vesicles were isolated from watermelon, or culture medium conditioned by human Caco-2 intestinal epithelial cells for 36h, using differential ultracentrifugation. Particles were subjected to nanoparticle tracking analysis to determine size. **(a)** Representative size distribution graph and **(b)** statistical comparison of watermelon EVs with Caco-2 exosomes. Data are shown as median ± interquartile range. (n=3-8; Mann-Whitney

test) **(c)** Representative images of watermelon EVs visualised by transmission electron microscopy demonstrating vesicular, cup-shaped morphology reminiscent of human exosomes analysed by this method. All scale bars represent 100nm. **(d)** Western blot analysis of positive (ALIX and CD63) and negative (CANX) markers for exosomes in EV lysates. Blots are representative of at least 3 individual experiments. **(e)** Relative protein levels (determined by mass spectrometry) of the targets of watermelon miRNAs which are enriched or depleted in WMEVs as compared to watermelon cells (Mann-Whitney test).

Supplementary Table 1

miRNA

Watermelon miRNA	*miFinder Rice miRNA*	*Log2 Fold change*
cla-miR171c	osa-miR171f-3p	6.80
cla-miR399g	osa-miR399c	3.59
cla-miR319a	osa-miR319b	2.95
cla-miR408	osa-miR408-3p	2.95
cla-miR390a	osa-miR390-3p	2.68
cla-miR160a	osa-miR160f-5p	2.66
cla-miR395a	osa-miR395a	1.62
cla-miR396a	osa-miR396b-3p	1.43
cla-miR156f	osa-miR156a	1.37
cla-miR167c	osa-miR167j	1.34
cla-miR2	osa-miR169o	1.25
cla-miR168a	osa-miR168b	1.09
cla-miR172	osa-miR172b	1.07
cla-miR166a	osa-miR166h-3p	1.00
cla-miR171a	osa-miR171h	-1.00
cla-miR319c	osa-miR159e	-1.08
cla-miR169c	osa-miR169e	-1.12
cla-miR164a	osa-miR164c	-1.46
cla-miR167a	osa-miR167a-5p	-1.76
cla-miR167a	osa-miR398b	-1.80
cla-miR396	osa-miR396c-5p	-2.04
cla-miR398a	osa-miR398a	-2.22
cla-miR169n	osa-miR169m	-2.37
cla-miR159a	osa-miR159a.1	-2.70
cla-miR393	osa-miR393b-5p	-2.70
cla-miR399a	osa-miR399d	-3.01
cla-miR169a	osa-miR169d	-3.76
cla-miR827	osa-miR827	-5.39

Protein

Accession	*Protein name*	*Log2 Fold change*
Cla021609	Proteasome subunit beta type	12.50
Cla014357	Glycyl-tRNA synthetase	12.00
Cla013914	Protein tolB	8.50
Cla006366	Beta-xylosidase 4	8.33
Cla008908	Proteasome subunit alpha type	8.28
Cla019264	Aspartyl aminopeptidase-like protein	6.90
Cla020011	Leucine aminopeptidase 1	6.48
Cla011114	chaperonin 2	6.00
Cla009244	ATP citrate synthase	6.00
Cla015537	Proteasome subunit alpha type	5.78
Cla022030	Alpha-1 4-glucan-protein synthase	5.44
Cla003640	Tripeptidyl peptidase ii	5.40
Cla017988	Pyridoxal biosynthesis lyase pdxS	5.34
Cla005148	Succinyl-CoA ligase	5.02
Cla002425	Homogentisate 1 2-dioxygenase	5.00
Cla000346	Lecithin cholesterol acyltransferase-like	5.00
Cla000562	Flavoprotein wrbA	4.75
Cla011434	Aminopeptidase N	4.67
Cla004613	Proteasome subunit alpha type	4.67
Cla021999	Proteasome subunit beta type	4.61
Cla020616	Progesterone 5-beta-reductase	4.50
Cla002881	Glutamate synthase	4.47
Cla022499	Glucose-6-phosphate isomerase	4.45
Cla018172	Proteasome subunit alpha type	4.33
Cla005762	Glycyl-tRNA synthetase	4.27
Cla022241	Proteasome subunit beta type	4.24
Cla013919	Proteasome subunit beta type	4.21
Cla008720	chaperonin 2	4.19
Cla021308	Proteasome subunit beta type	4.12
Cla014298	Proteasome subunit alpha type	4.10
Cla004612	Proteasome subunit alpha type	4.01
Cla007766	26S proteasome non-ATPase regulatory subunit 12	4.00
Cla018547	Alpha-1 4-glucan-protein synthase	4.00
Cla004347	1-phosphatidylinositol-4 5-bisphosphate phosphodiesterase	4.00
Cla007056	Alcohol dehydrogenase C	4.00
Cla015416	NMDA receptor regulated 1-like	4.00
Cla020201	Ribosomal protein L26 component of cytosolic 80S ribosome and 60S large subunit	4.00
Cla012168	Proteasome subunit alpha type	3.93
Cla004677	heat shock protein	3.83
Cla011286	Asparaginyl-tRNA synthetase	3.78

Supplementary Table 1. Fold change in miRNAs and proteins in watermelon extracellular vesicles compared to watermelon cells. Watermelon cells (1,000xg pellet) and extracellular vesicles (EVs; 100,000xg pellet) were isolated (n=3) using differential

sequential ultracentrifugation and their miRNA profiled using the miScript Rice miFinder qPCR array. 48 of the 84 miRNAs included in the array passed the quality control measures (<40 Ct and <2°C Tm difference between samples); of these, 24 were increased >2-fold and 9 were decreased >2-fold in watermelon EVs compared to source cells. The remaining 11 miRNAs were unchanged (less than a 2-fold ΔΔCt difference between samples). Proteins extracted from watermelon cells and extracellular vesicles (EV) were identified using mass spectrometry. The top 40 proteins most increased in EVs compared to the source cells are presented.

Supplementary Table 2

Accession	No. Plant Species where Protein is Present in EVs	Protein Name	Watermelon EVs vs Cells (Fold-change)
Cla012175	4	Triosephosphate isomerase	18.22
Cla017822	4	V-type proton ATPase subunit G	17.21
Cla007587	4	Malate dehydrogenase	8.1
Cla007792	4	Actin	5.57
Cla015847	4	Peptidyl-prolyl cis-trans isomerase	4.56
Cla011437	4	Aquaporin 1	4.05
Cla018715	4	Outer membrane lipoprotein Blc	4
Cla020983	4	Vesicle-associated membrane protein 7C	3
Cla012184	4	Pyrophosphate-energized vacuolar membrane proton pump family protein	2.53
Cla010547	4	Adenosylhomocysteinase	2.02
Cla005204	4	Heat shock protein 90	2
Cla001345	4	Vesicle-associated protein	2
Cla023344	4	Sodium/calcium exchanger family protein	2
Cla016803	4	Profilin	2
Cla019719	4	Tubulin beta chain	1.77
Cla018367	5	Enolase	1.45
Cla012806	4	Nucleoside diphosphate kinase	1.01
Cla003708	4	Fasciclin-like arabinogalactan protein 3	1
Cla023447	4	Catalase	-1.15

Supplementary Table 2. Proteins common to the EVs of multiple plant species. The proteins identified in WMEVs in this study were compared to the proteins present in EVs from grape (Molecular Therapy 2013 21:1345), grapefruit (Molecular Therapy 2014 22:522), lemon (Oncotarget 2015 6:19514) and *Arabidopsis thaliana* (Plant Physiology 2017 172:728). Table shows proteins present in 4 or more species.

Supplementary Figure 2

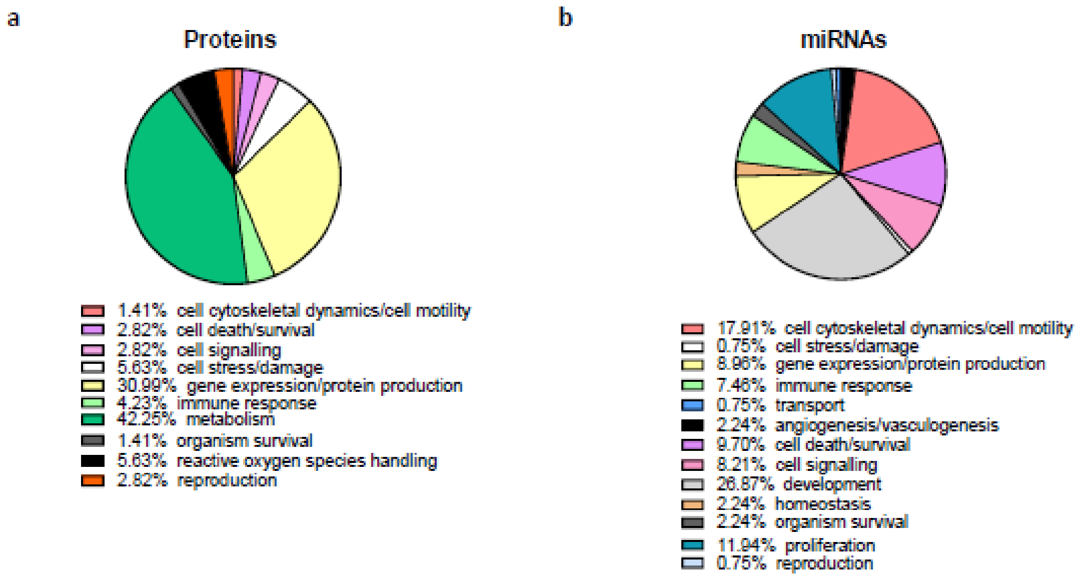

Supplementary Figure 2. Bioinformatics analysis of watermelon EV constituents - prediction of WMEV function in mammalian cells; determination of the potential function of WMEV (a) proteins or (b) miRNAs (via their predicted targets) in mammalian cells was conducted using IPA Ingenuity functional enrichment analysis. Significantly enriched functions were grouped by 'mother' function (i.e., cell signalling). The Fisher's Exact test was used to identify enrichment. Multiple testing was controlled for using the Benjamini-Hochberg Procedure with a cutoff of $q<0.05$ for significance.

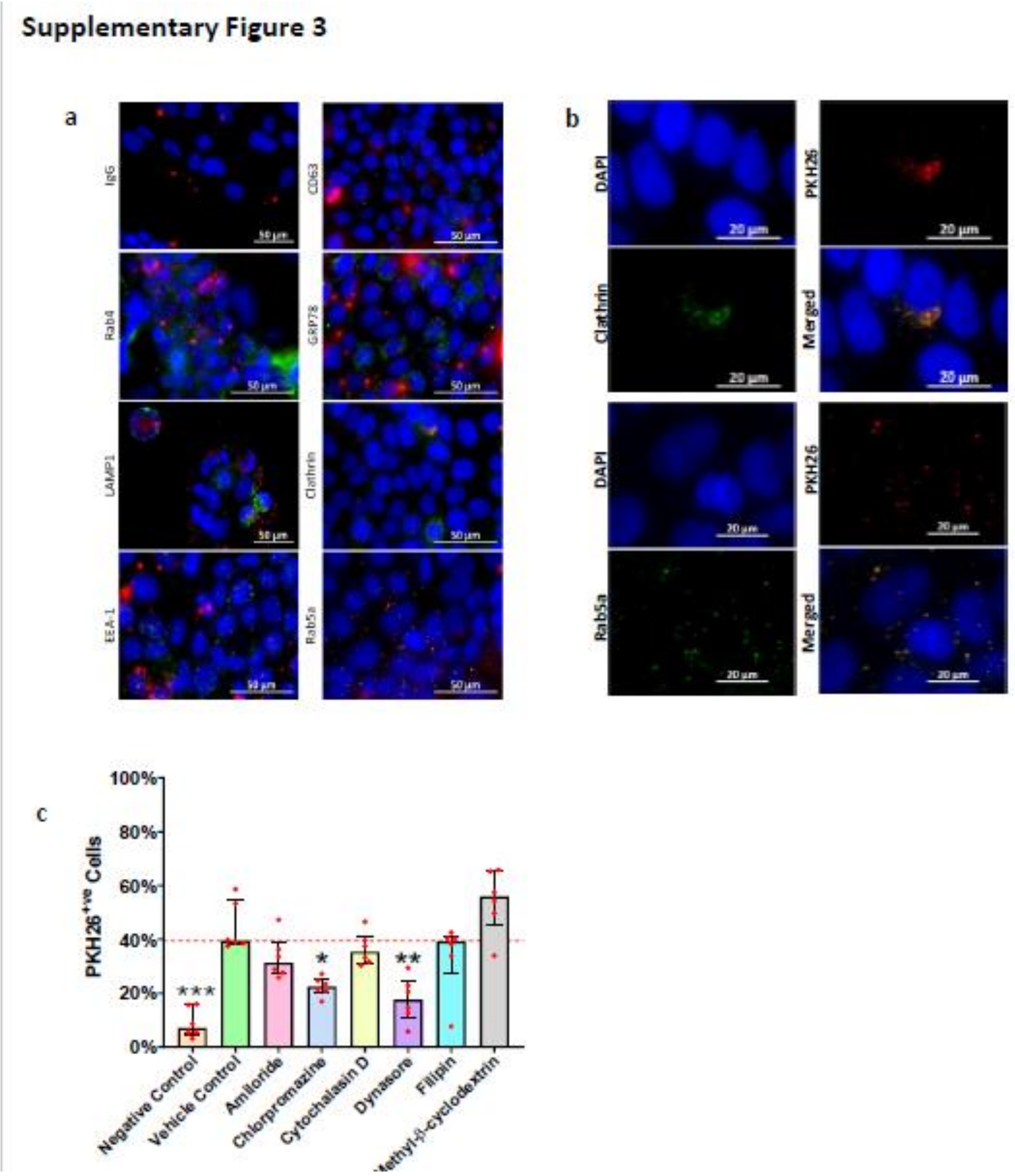

Supplementary Figure 3. Watermelon extracellular vesicles are internalised into human intestinal epithelial cells by clathrin-mediated endocytosis.

(a) Undifferentiated Caco-2 cells were incubated for 180 minutes with PKH26-labelled WMEV (red; 2.5 x 10^{10} EVs/ml) then fixed and nuclei stained with DAPI (blue) and for CD63, Rab4, GRP78, LAMP1, clathrin, EEA-1 or Rab5a. Secondary antibodies were Alexa Flour 647 and have been false coloured green. Images are representative of at least 3 individual experiments. Higher magnification and non-merged images of clathrin and Rab5a staining are shown in **(b)**. **(c)** Differentiated Caco-2 cells (n=6) were treated (apically and basally) for 1 hour with either uptake inhibitors or the appropriate concentration of DMSO or methanol (vehicle control) then apically incubated with 2.5 x 10^{10} PKH26-labelled WMEVs/ml for 180 minutes. As controls, cells were incubated with the equivalent volume of dye control or DMSO/methanol in the absence of WMEVs. PKH26 fluorescence was measured using flow

cytometry. Kruskal-Wallis test with Dunn’s multiple comparisons test; * = p<0.05, ** = p<0.01. Data are presented as median ± interquartile range.

Supplementary Figure 4

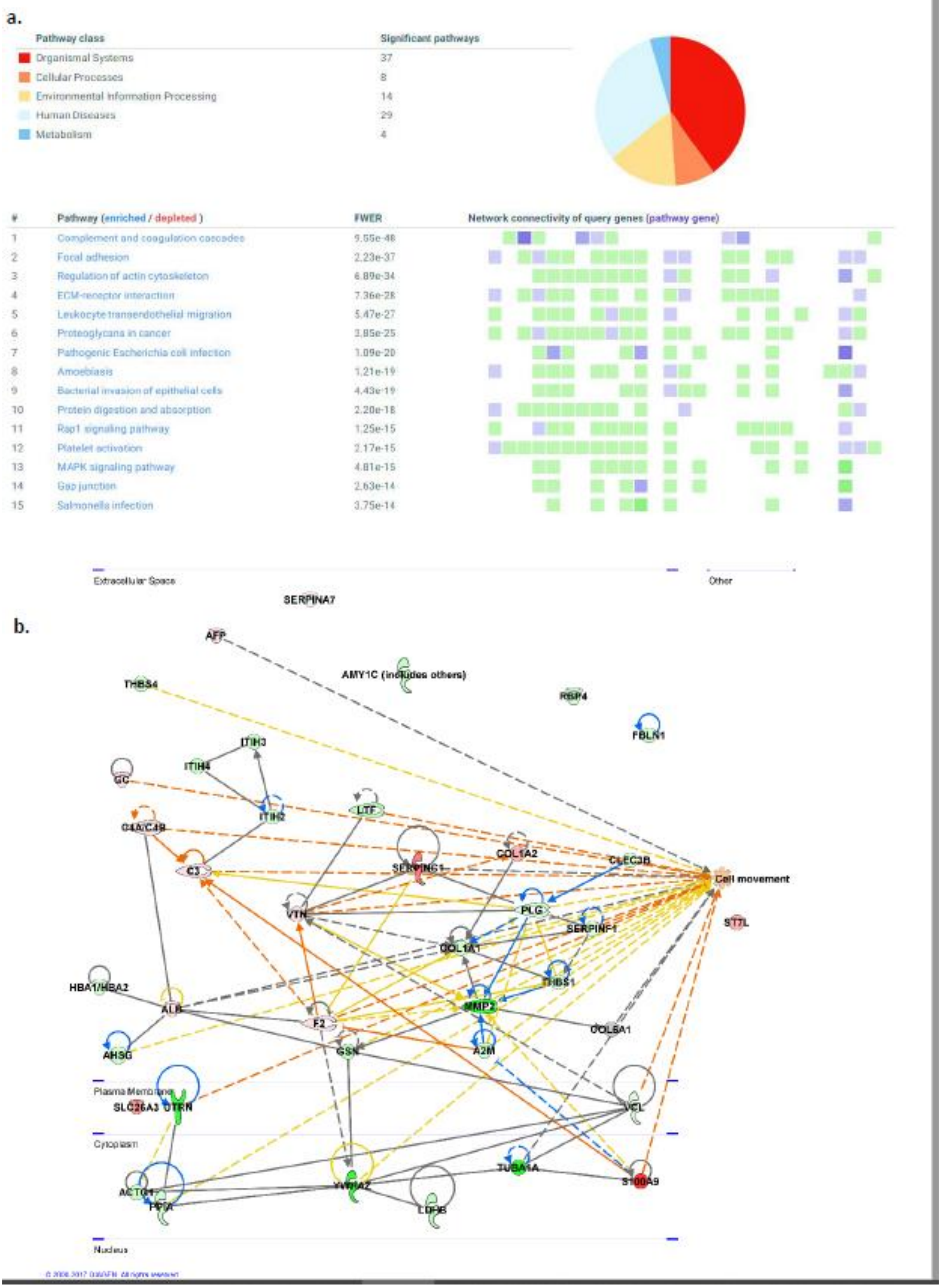

Supplementary Figure 4. Predicted impact of the Caco-2 basal secretome altered in response to watermelon extracellular vesicle treatment. Proteins in the basal medium from Caco-2 cells exposed apically to watermelon extracellular vesicles for 4h were analysed for (**a**) pathways crosstalk and (**b**) by network and predicted downstream function analysis, which predicted an increase in cell movement. Red and green indicate upregulation and downregulation in the dataset respectively. Orange and blue indicate predicted stimulation and inhibition respectively. Grey indicates no predicted change in activity.

Supplementary Figure 5

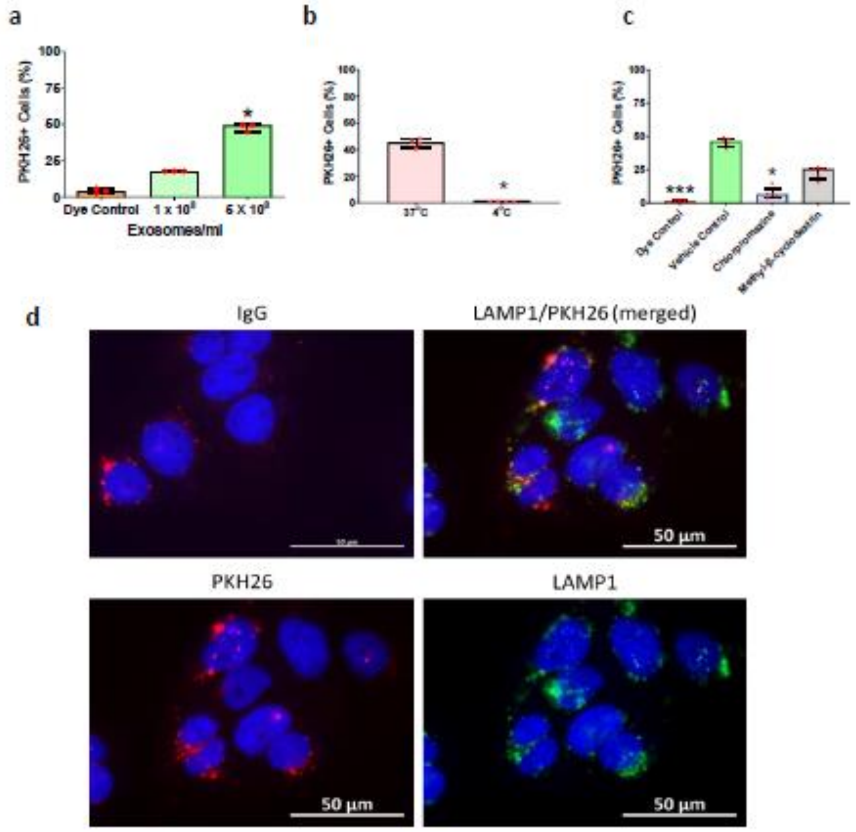

Supplementary Figure 5. Uptake of Caco-2 EVs into BeWo cell LAMP1-positive late endosomes/lysosomes by clathrin-mediated endocytosis. BeWo cells were incubated with (**a**) dye control, 1 x 10^8 or 5 x 10^8 PKH26-labelled Caco-2 EVs/ml for 240 minutes, or with 5 x 10^8 EVs/ml for 240 minutes at either (**b**) 4°C or 37°C (n=4), or (**c**) at 37°C following 45 minute pre-treatment with chlorpromazine (20µg/ml) or methyl-β-cyclodextrin (13mg/ml) (n=5; significance given is with respect to vehicle control). The percentage of PKH26^{+ve} cells was measured using flow cytometry. (**d**) Alternatively, cells were processed for fluorescence microscopy and stained with an antibody against LAMP1, a marker of late endosomes and lysosomes. Mann-Whitney test (**a-b**), Kruskal-Wallis test with

Dunn's multiple comparisons test (**b**), * = $p<0.05$, *** = $p<0.001$. Data are presented as median ± interquartile range.
